# Developing and validating predictive decision tree models from mining chemical structural fingerprints and high–throughput screening data in PubChem

**DOI:** 10.1186/1471-2105-9-401

**Published:** 2008-09-25

**Authors:** Lianyi Han, Yanli Wang, Stephen H Bryant

**Affiliations:** 1 National Center for Biotechnology Information, National Library of Medicine, National Institutes of Health, Bethesda, MD 20894, USA

## Abstract

**Background:**

Recent advances in high-throughput screening (HTS) techniques and readily available compound libraries generated using combinatorial chemistry or derived from natural products enable the testing of millions of compounds in a matter of days. Due to the amount of information produced by HTS assays, it is a very challenging task to mine the HTS data for potential interest in drug development research. Computational approaches for the analysis of HTS results face great challenges due to the large quantity of information and significant amounts of erroneous data produced.

**Results:**

In this study, Decision Trees (DT) based models were developed to discriminate compound bioactivities by using their chemical structure fingerprints provided in the PubChem system . The DT models were examined for filtering biological activity data contained in four assays deposited in the PubChem Bioassay Database including assays tested for 5HT1a agonists, antagonists, and HIV-1 RT-RNase H inhibitors. The 10-fold Cross Validation (CV) sensitivity, specificity and Matthews Correlation Coefficient (MCC) for the models are 57.2~80.5%, 97.3~99.0%, 0.4~0.5 respectively. A further evaluation was also performed for DT models built for two independent bioassays, where inhibitors for the same HIV RNase target were screened using different compound libraries, this experiment yields enrichment factor of 4.4 and 9.7.

**Conclusion:**

Our results suggest that the designed DT models can be used as a virtual screening technique as well as a complement to traditional approaches for hits selection.

## Background

High-throughput screening (HTS) is an automated technique and has been effectively used for rapidly testing the activity of large numbers of compounds [1-3]. Advanced technologies and availability of large-scale chemical libraries allow for the examination of hundreds of thousands of compounds in a day via HTS. Although the extensive libraries containing several million compounds can be screened in a matter of days, only a small fraction of compounds can be selected for confirmatory screenings. Further examination of verified hits from the secondary dose-response assay can be eventually winnowed to a few to proceed to the medicinal chemistry phase for lead optimization [4,5]. The very low success rate from the hits-to-lead development presents a great challenge in the earlier screening phase to select promising hits from the HTS assay [4]. Thus, the study of HTS assay data and the development of a systematic knowledge-driven model is in demand and useful to facilitate the understanding of the relationship between a chemical structure and its biological activities.

In the past, HTS data has been analyzed by various cheminformatics methods [6-17], such as cluster analysis[10], selection of structural homologs[11,12], data partitioning [13-16] etc. However, most of the available methods for HTS data analysis are designed for the study of a small, relatively diverse set of compounds in order to derive a Quantitative Structure Activity Relationship(QSAR) [18-21] model, which gives direction on how the original collection of compounds could be expanded for the subsequent screening. This "smart screening" works in an iterated way for hits selection, especially for selecting compounds with a specific structural scaffold [22]. With the advances in HTS screening, activity data for hundreds of thousands' compound can be obtained in a single assay. Altogether, the huge amount of information and significant erroneous data produced by HTS screening bring a great challenge to computational analysis of such biological activity information. The capability and efficiency of analysis of this large volume of information might hinder many approaches that were primarily designed for analysis of sequential screening. Thus, in dealing with large amounts of chemicals and their bioactivity information, it remains an open problem to interpret the drug-target interaction mechanism and to help the rapid and efficient discovery of drug leads, which is one of the central topics in computer-aided drug design [23-30].

Although the (Quantitative) Structure Activity Relationship-(Q)SAR has been successfully applied in the regression analysis of leads and their activities [18-21], it is generally used in the analysis of HTS results for compounds with certain structural commonalities. However, when dealing with hundreds of thousands of compounds in a HTS screening, the constitution of SAR equations can be both complicated and impractical to describe explicitly.

Molecular docking is another widely used approach to study the relationship between targets and their inhibitors by simulating the interactions and binding activities of receptor-ligand systems or developing a relationship among their structural profiles and activities[31,32]. However, as it takes the interactions between the compounds and the target into consideration, it has been widely used for virtual screening other than to extract knowledge from experimental activities.

Decision Tree (DT) is a popular machine learning algorithm for data mining and pattern recognition. Compared with many other machine learning approaches, such as neural networks, support vector machines and instance centric methods etc., DT is simple and produces readable and interpretable rules that provide insight into problematic domains. DT has been demonstrated to be useful for common medical clinical problems where uncertainties are unlikely [33-37]. It has been applied to some bioinformatics and cheminformatics problems, such as characterizations of Leiomyomatous tumour[38], prediction of drug response[39], classification of antagonist of dopamine and serotonin receptors[40], virtual screening of natural products[41].

In this study, we propose a DT based model to generalize feature commonalities from active compounds tested in HTS screening. We utilized DT as the basis to develop the model because it has been successfully applied in many biological problems, and it is able to generate a set of rules from the active compounds which can then be used for filtering the untested compounds that are likely to be active in the biological system of interest. Moreover, it has the capability to handle the arbitrary degree of non-linear structurally diversified compounds.

Many elegant algorithms for building decision tree models have been introduced and applied in real life problems, and C4.5[42] is one of the best known programs for constructing decision trees. In this work, the DT based model was developed on the basis of the Decision Tree C4.5 algorithm[42]. The representation of the molecular structures is described by the PubChem fingerprint system. The DT based model was further examined by four assays deposited in the PubChem Bioassay Database, the HTS assay for 5-Hydroxytryptamine Receptor Subtype 1a (5HT1a) antagonists(PubChem AID:612), HTS assay for 5HT1a agonists(PubChem AID 567), and two other assays with PubChem AID 565 and 372 for screening the HIV-1 RT-RNase H inhibitors. The results of 10-fold Cross Validation (CV) over these HTS assays suggest the self-consistency of the DT models. Since a model simply provides the rules based on the profiles of active compounds in a specific HTS assay, the computationally generated models were further examined using two HTS assays which tested the same HIV RNase target, but used different compound libraries and were performed independently by two individual research laboratories. Our results suggest that these developed models could be used to validate HTS assay data for noise reduction and to identify hits through virtual screening of additional and larger compound libraries.

## Results and discussion

### Development of DT model

In this study, four DT models were developed for activity data contained in PubChem bioassay AID 612, 567, 565 and 372 respectively, where compounds were screened for various activities against several protein targets (Table 1).

**Table 1:** HTS assays analyzed in this study

**Protein Target**	**The role of active compounds**	**PubChem Bioassay AID No.**	**Number of Compounds tested**	**Number of active compounds identified**
5-Hydroxytryptamine Receptor Subtype 1a	agonist	567	64,906	366
	antagonist	612	61,606	416
HIV-1 reverse transcriptase associated ribonuclease H	inhibitor	565	65,216	1,250
		372	99,768	770

Model fitting accuracies are used to examine whether the proposed data model can handle the complex data and whether the chemical fingerprint descriptors are sufficient for model development. As shown in Table 2, the model fitting accuracies of the four DT models were in the range of 98.6% to 99.8%. This suggested that the DT based models are able to fit the majority of the HTS data for the model generalization. Sensitivity and specificity of each developed model, which indicate the ratio of the active and inactive compounds that can be successfully learned by the DT model, are also reported in Table 2. The sensitivities of the models built for AID 612, AID 567, AID 565 and AID 372 are 86.5%, 98.9%, 90.2% and 83.1% respectively, while the specificities are all greater than 98%. The model fitting accuracies for both active and inactive compounds suggest strong feature-activity relationship among compounds tested in the HTS screenings. The small fraction of misrecognized compounds might result from the HTS data noise, the discrepancy of bioactivities observed from the compounds with same or similar chemical structures, or the competition with the overwhelming inactive compounds

**Table 2:** Recognition rate of Decision Tree models

		**Active compounds**			**Inactive compounds**					
						
**Bioassay**	**PubChem Assay ID**	**TP**	**FN**	**Sensitivity**	**TN**	**FP**	**Specificity**	**Overall accuracy**	**MCC**	**Model Complexity (Number of Nodes/Number of Leaves/Number of Features)**
5HT1a agonist	567	362	4	98.9%	64,394	146	99.8%	99.8%	0.84	(321/161/149)
5HT1a antagonist	612	360	56	86.5%	60,909	281	99.5%	99.5%	0.70	(1135/568/261)
HIV-1 RT RNase H inhibitor	565	1,128	122	90.2%	63,070	896	98.6%	98.4%	0.70	(3003/1502/412)
HIV-1 RT RNase H inhibitor	372	640	130	83.1%	98,463	535	99.5%	99.3%	0.67	(2511/1256/370)

TP = true positives, the number of correctly recognized active compounds;

FN = false negative, the number of active compounds that the model is unable to recognize;

TN = true negative, the number of inactive compounds that successfully recognized by the model;

FP = false positive, the number of inactive compounds that the model is unable to recognize.

As shown by the comparison of the sensitivity and its corresponding specificity for each individual DT model, the sensitivity is usually lower than the specificity and contributes less to the overall accuracy. One possible explanation is the existence of the data imbalance issue. Among the HTS data analyzed in this study, the ratio between the number of active compounds and that of inactives are ranged from 1:51~1:176. Thus comparing to the active compounds, the rules could be easily generalized for inactive compounds when the chance of pattern reoccurrence is higher. Data Imbalance problem is common in the high throughput screening assay data. One HTS assay could have tens of thousands of compounds tested and only yield few dozens of hits. Due to this problem, the specificity becomes less objective in performance evaluation. Therefore, we also use the Matthews Correlation Coefficient (MCC) [43] as additional measure to evaluate the model's performance.

MCC took both sensitivity and specificity into account and it is generally used as a balanced measure in dealing with data imbalance situation. As shown in Table 2, MCC values fall in the range of 0.67~0.84 for the four HTS assays, which again suggests the satisfactory performance of the model training and indicates that the self recognition of the model is not random.

### Model validation by self-consistency test

The validation of the DT based models and self-consistency test were performed by 10-fold cross validation (CV) method, in which the compound dataset tested in one HTS assay was randomly split into 10 folds. These models were set up using 9 randomly selected folds, and prediction was done on the remaining fold.

The 10-fold CV results are given in Table 3. The overall validation accuracies of all DT models ranged from 96.9% to 98.9%. While the sensitivities of the models built for AID 612, AID 567, AID 565 and AID 372 are 64.5%, 80.5%, 75.2% and 57.2% respectively, where the specificities were 99.1%, 99.0%, 97.3% and 98.9% respectively. The more than 96% overall accuracies of the four DT based models suggest overall good performance and the CV analysis validates the reliability of the DT based models.

**Table 3:** Performance evaluation of Decision Tree models by 10 fold Cross Validation.

		**Active compounds**			**Inactive compounds**				
					
**Bioassay**	**PubChem Assay ID**	**TP**	**FN**	**Sensitivity**	**TN**	**FP**	**Specificity**	**Overall accuracy**	**MCC**
5HT1a agonist	567	295	71	80.5%	63913	627	99.0%	98.9%	0.50
5HT1a antagonist	612	268	148	64.5%	60656	534	99.1%	98.9%	0.46
HIV-1 RT RNase H inhibitor	565	940	310	75.2%	62269	1698	97.3%	96.9%	0.50
HIV-1 RT RNase H inhibitor	372	441	329	57.2%	97923	1075	98.9%	98.6%	0.40

TP = true positives, the number of correctly recognized active compounds;

FN = false negative, the number of active compounds that the model is unable to recognize;

TN = true negative, the number of inactive compounds that successfully recognized by the model;

FP = false positive, the number of inactive compounds that the model is unable to recognize.

The sensitivity and specificity values given here represent the classification accuracies for the active and inactive compounds respectively. The sensitivity is lower than specificity to a certain extent. For example, the DT model for the 4HTa antagonist activity data demonstrates 64.5% sensitivity but 99.1% specificity. From the evidence given in the previous section, imbalanced data, data noise and data discrepancy could again account for the lower sensitivities. Moreover, as about 90% percent of the data used for training during the cross validations, the generalization ability of the active compound dataset became easily affected due to the limited sample size as compared to that of the inactive compound dataset.

The learning capability of the DT model could also be affected by the way the model was trained, such as the minimum count of compound instances required for a decision node. However, it primarily depends on the datasets used for training. Although the imbalanced active and inactive compound datasets have an effect on the performance of the 10-fold CV, our results still show that the models are self-consistent. In addition, compounds and their activity data in HTS screens are able to converge toward a discrimination model with encouraging accuracies. In addition, the MCC values ranged from 0.4 to 0.5, again indicating the potential of the models to identify potential hits.

### Application of DT models to select potential active compounds

In this study, independent evaluation of the DT based models was attempted by using two HTS assays, PubChem AID 565 and 372, which were aimed at identifying HIV-1 RT RNase H inhibitors.

Comparison of the compound libraries of these two HTS assays were first performed, demonstrating the extent of similarity of the active compounds between the two assays. By using Tanimoto coefficient[11,12] as a measurement for the compound similarities, there are only six active compounds that were found to be similar with Tanimoto coefficient threshold of 95%. This suggested the overlap of the active compounds in these two assays was very limited. It maybe of interest to investigate whether the DT model built with one compound set can be used to filter out hits identified with another assay where a different compound library was screened. To this end, DT models of these two HTS assays were first developed independently and then each model was applied to classify the compounds screened by the other assay. An enrichment factor, which simply describes the proportion of active compounds from any given collection compared with randomly picked compounds [44], was calculated as assessment for the classification performance of each model.

Assume N compounds are tested in a HTS assay sample where A compounds have been experimentally verified as bioactive. By virtual screening, which is the activity classification using DT model in this study, N_s _compounds are predicted as active and among these N_a _belongs to the group of known bioactive compounds. A randomly picked sample will on average contain AN_s_/N active molecules. Therefore, the formula for calculating the enrichment factor is N_a_N/N_s_A.

The enrichment factors for cross dataset prediction of HIV RNase H inhibitors of AID 372 and 565 are 4.4 and 9.7 respectively. From the virtual screening point of view, which is focusing on selecting the true hits while excluding the false positives as much as possible, the results suggest that the model derived from these two bioassays have certain generalization abilities to increase the odds of selecting true hits.

On the other hand, the sensitivities of the DT models based on data sets AID 372 and 565 are 0.4% and 6.9%, and the specificities are 98.5% and 99.9% respectively, which yield corresponding MCC value of 0.03 and 0.04, apparently the sensitivities and MCC values in this experiment are "poor" comparing to the cross validation study. This is not surprising, and indeed is well expected as the dramatic chemical structural differences between the two data sets (AID 372 and AID 565), and the models derived from the individual datasets may carry overwhelming localization features that might not be largely applicable to each other. This also leaves the gap to be filled in for a robust statistical model, better representation of physical chemical properties, enlarged and diversified dataset, and enhanced quality of the experimental accuracy in the future.

Nevertheless, this preliminary test using DT model as virtual screening technique yields encouraging enrichment for selection of active compounds when applied to another HTS activity dataset. It suggests that, despite of the very low similarities between the active compounds from the two HTS assays, certain common profiles of the active compounds can be extracted using the DT model, which can ultimately be very useful for virtual screening tasks.

## Conclusion

In this study, we use derived DT models based on structural fingerprints of compounds to select biologically interesting compounds from HTS assay dataset. Four HTS assays were analyzed to determine to what extent the designed models can be applied to the compound libraries of an unknown domain. Our results suggest that the DT based models can be successfully used to derive common characteristics from HTS data, and the models can serve as filters to facilitate the selection of compounds against the same target. These DT models could also be used to eliminate HTS hits arising from data noise or those lacking statistical significance.

The development of the model is a learning process. Thus, the potential of the developed model is limited to the known active compounds and the properties used for training, and limited to the distribution of the compound collection to which the model is applied. With the growth in the number of compounds to be screened and the improvement over data quality produced with HTS assay, a more robust model could be developed with increased ability for selecting biologically interesting small molecules from a diverse compound library.

## Methods

### Datasets

There are over 500 assays deposited in the PubChem Bioassay database as of May 1^st^, 2007. About 200 of them have protein targets. In this study, four HTS assays were selected from the PubChem bioassay database. The criteria for the selection were that a substantial number of compounds have been tested in one assay and that the number of active compounds is in the hundreds to demonstrate statistical significance. These HTS assays are 5-Hydroxytryptamine Receptor Subtype 1a (5HT1a) antagonists with PubChem BioAssay AID 612, HTS assay for screening 5HT1a agonists with AID 567, and PubChem bioassays AID 565, AID 372 for screening HIV-1 RT-RNase H inhibitors. The number of structurally distinct compounds for these four bioassays and the number of active and inactive compounds are summarized in Table 1. Compound bioactivities outcomes have been described in a binary form, active and inactive, as specified by the assay depositor, and were retrieved from PubChem BioAssay database.

### PubChem Fingerprint System

The numerical understanding of chemical structures is described by a binary substructure fingerprint generated by the PubChem Fingerprint System.

A substructure is a fragment of a chemical structure, such as a type of ring system, atom pairing, or atom environment (nearest neighbours). A fingerprint is an ordered list of binary (1/0) bits. Each bit represents a Boolean determination of the presence of a fragment of a chemical structure. The PubChem fingerprint has a total of 881 bits and is composed of 7 sections such as Hierarchic Element Counts, chemical rings, and simple atom pairs, simple atom nearest neighbours, detailed atom information and two sections of SMARTS patterns. A detailed description and the full list of fingerprint bits can be accessed at .

The 2D structure of each compound was used to generate a binary substructure fingerprint.

### Decision Tree based Filtering Method

DT is an acyclic graph in which its interior vertices specify testing of a single attribute of a feature vector and its leaves indicate the class of the decision [45-47]. It was constructed by recursively splitting the sample set, with each subset giving rise to one new vertex connected with an edge to its parent. This procedure continues until all samples at each leaf belong to the same class. The working flow of DT is similar to a logical tree structure that starts from the topmost node, and every decision of the node determines the direction of next node movement until the end of the tree branch node is reached. In this study, we performed DT analysis by utilizing the C4.5 core library [42].

As the filtering system is designed to choose the compounds of interest with certain features' commonalities to those compounds considered as active in the HTS assay, the binary representation of the activity outcomes were used to categorize these compounds. The structural fingerprint of every compound was processed by the PubChem fingerprints system subsequently for numerical description of the dataset for model training. To derive the models, the logical decision tree is then examined for error pruning, which is the removal of branches that are deemed to provide little or no gain in statistical accuracy of the model.

### Model Self-consistency evaluation

The model self-consistency evaluation is performed using the 10 fold CV approach. As the HTS data are usually diversely distributed and not error free, the CV evaluation of the DT model is subjected to representatives from both the compounds used for training and for testing. Thus, for the balance between the computation cost and the evaluation of the model generalization ability, the 10 fold CV approach is chosen to assess the self-consistency of the model [48].

Under the assumption that the distribution of different subsets from one HTS assay is approximately equal, the quality of the model can be proven if the model built on the top of a portion of the data can be generalized to others during the self-consistency evaluation.

### Measurement of accuracies

Model accuracy is measured by sensitivity, specificity, and a combined parameter called "overall accuracy." The sensitivity and specificity are defined as the following:

Sensitivity=TP(TP+FN),Specificity=TN(TN+FP),

where the true positive (TP) is the number of compounds correctly predicted as active, false negative (FN) is the number of compounds incorrectly predicted as inactive, true negative (TN) is the number of compounds correctly predicted as inactive, and false positive (FP) is the number of compounds incorrectly predicted as active. Thus, the overall accuracy is defined as $Overall\_Accuracy=\frac{TP+TN}{TP+FN+TN+FP}\times 100\%$.

In addition to compute sensitivity and specificity, to further evaluate the classification performance on a dataset containing imbalanced active and inactive compounds, Matthews correlation coefficient (MCC)[49] is also calculated as given by the following equation

$$MCC=\frac{TP\cdot TN-FN\cdot FP}{\sqrt{\left(TP+FN)(TP+FP)(TN+FN)(TN+FP\right)}}$$

MCC ranges from -1 to 1, and suggests the randomness of the model.

### Use of DT models to select compounds of interest

The best model is optimized by pruning the decision tree to minimize the classification errors. With the knowledge learned from the tested compounds and their bioactivities, one can apply these trained rules to filter new compounds that are likely to be active. In addition to the validation of the DT models with CV approach, application of models for the prediction of bioactivity classification have been attempted by using two HTS assays for identifying HIV-1 RT RNase inhibitors. Both of these two HTS assays are designed for the screening of the HIV-1 RT RNase H target. In spite of the differences in the design of HTS assays and in the selection of compound libraries, the underlying knowledge of those inhibition compounds was assumed to be similar or interpretable from one another. To this end, the optimized DT models of these two HTS assays were developed independently and were applied to examine the other compound collection as virtual experiments for identifying potential inhibitors.

### Computation Software

The implementation of the Decision Tree-based models was based on PubChem Fingerprint System, OpenEye OEChem C++ library, NCBI C++ toolkit library, and the C4.5 core library.

## Authors' contributions

All authors participated in development of the methods, discussions and preparation of the manuscript.
